# Development of a Modified Textbook Outcome in Evaluating Robot‐Assisted Middle Pancreatectomy: A Real‐World Study of RMP Surgery in a High‐Volume Pancreatic Disease Center

**DOI:** 10.1002/cam4.71542

**Published:** 2026-01-30

**Authors:** Jingfeng Li, Lihan Qian, Zhiwei Xu, Xinjing Wang, Wei Xu, Xiaxing Deng, Chenghong Peng, Baiyong Shen, Yusheng Shi

**Affiliations:** ^1^ Department of General Surgery, Pancreatic Disease Center, Ruijin Hospital Shanghai Jiao Tong University School of Medicine Shanghai China; ^2^ Shanghai Key Laboratory of Pancreatic Neoplasms Translational Medicine Shanghai China; ^3^ Research Institute of Pancreatic Diseases, Shanghai Jiao Tong University School of Medicine Shanghai China; ^4^ State Key Laboratory of Oncogenes and Related Genes Shanghai China; ^5^ Institute of Translational Medicine, Shanghai Jiao Tong University Shanghai China

**Keywords:** post‐operative pancreatic fistula, robot‐assisted middle pancreatectomy, textbook outcome

## Abstract

**Objective:**

We aimed to, for the first time, assess the value of modified textbook outcome (mTO) in robot‐assisted middle pancreatectomy (RMP) procedures.

**Summary Background Data:**

Pancreatic fistula remains to be the major complication after RMP. Textbook outcome (TO) is introduced to capture the most desirable surgical outcomes. The value of TO in RMP surgery remains unknown.

**Methods:**

All patients who underwent RMP in our center from 2010 to 2023 were enrolled in the study. Baseline characteristics, operative outcomes, and oncological outcomes were collected and analyzed. Textbook outcome was calculated separately for each patient and analyzed.

**Results:**

The mTO was defined by the absence of modified post‐operative pancreatic fistula (mPOPF), postpancreatectomy hemorrhage (PPH), severe complications (Clavien–Dindo ≥ III), readmission, and in‐hospital mortality (IHM). The overall mTO rate and mPOPF rate of 209 patients were 73.68% and 15.79%, respectively. Patients who achieved modified textbook outcomes have shorter post‐operative hospitalization days (median (IQR), 17 (9) vs. 34 (26), *p* < 0.001). Passing the learning curve leads to a reduction of the mPOPF rate and an increase of the mTO rate.

**Conclusions:**

Modified textbook outcome is a practical metric for evaluating ideal surgical outcomes in RMP surgery. Follow‐up multi‐center clinical research is necessary to evaluate this indicator even further.

Typically, the incidence of specific postoperative complications is used to assess the safety of an operation or to predict patient outcomes. For example, we used the incidence of postoperative pancreatic fistula (POPF) to evaluate the success of pancreatectomy. However, a single parameter does not reflect the multidimensional aspects of the entire surgical procedure [1, 2]. Thus, the textbook outcome (TO) was introduced as a comprehensive single indicator that represents the most desirable surgical outcomes. The TO is realized in terms of the all‐or‐none principle; specifically, the ideal surgical outcome is achieved when all of the prespecified requirements for the TO are fulfilled [3]. According to research conducted by van Roessel in 2020, the TO following pancreatoduodenectomy (PD) and distal pancreatectomy (DP) was defined as the absence of postoperative pancreatic fistula, bile leakage, postpancreatectomy hemorrhage (all ISGPS grades B/C), severe complications (Clavien–Dindo grade ≥ III), readmission, and in‐hospital mortality [4].

Middle pancreatectomy (MP) is a surgical procedure indicated for the radical treatment of benign or non‐cancerous lesions located in the neck or proximal body of the pancreas. Compared with pancreaticoduodenectomy (PD) or distal pancreatectomy (DP), MP causes less trauma, and less pancreatic loss allows for better functional preservation and quality of life (QOL). The first MP was performed by Guillemin and Bessot in 1957 [5]. The first laparoscopic middle pancreatectomy (LMP) and the first robot‐assisted middle pancreatectomy (RMP) procedures were reported by Baca and Bokan in 2003 and by Giulianotti et al. in 2010, respectively [6, 7]. The MP procedure possesses strict criteria, including tumor location, pathology, and vascular adjacency [8]. Several previous studies, including those previously conducted at the author's pancreatic center, have demonstrated the safety and feasibility of both open and robot‐assisted middle pancreatectomy [9, 10, 11, 12, 13, 14]. As the first high‐volume pancreatic center in China to begin and perform the greatest number of RMP surgeries, we published, for the first time, an article regarding the learning curve of RMP surgery in 2019 [14].

POPF remains the most frustrating postoperative complication after MP. Previous studies have reported a 20%–50% incidence of POPF after surgery [9, 10, 11, 12], and the overall POPF rate was observed to be 32% in our previous study [14]. Such a high rate of POPF can result in a lack of confidence when surgeons are deciding to select MP. Therefore, the aim of this study is to investigate whether stereotypes about MP can be changed by introducing the TO indicator. Due to the differences in the nature of MP versus PD and DP procedures, we proposed a modified TO definition. Additionally, we conducted a systematic review of the TO metrics of previous MP procedures performed in our high‐volume pancreatic center.

## 1 Materials and Methods

### 1.1 Study Design and Data Collection

Patients who underwent robot‐assisted middle pancreatectomy (RMP) were enrolled in this study. The data were obtained from the database of the Department of General Surgery, Pancreatic Disease Center, Ruijin Hospital Shanghai Jiao Tong University School of Medicine, Shanghai, China from December 2010 to December 2023. Patients with missing data on 1 or more of the requirements for TO were excluded.

This study was approved by the Ruijin Hospital Ethics Committee. All of the patients were asked to provide informed consent to ensure that they agreed to undergo the operation and to the use of data we collected before and after surgery. The study was performed according to the Strengthening the Reporting of Observational Studies in Epidemiology (STROBE) guidelines [15]. The requirement for informed consent was waived due to the retrospective nature of the study. All of the patients underwent routine preoperative computed tomography (CT) and magnetic resonance imaging (MRI). Endoscopic ultrasonography (EUS) was performed if the type of tumor was unclear. The tumor was diagnosed and the surgical protocol was determined by our multidisciplinary team (MDT). All of these surgeries were performed by the same group of surgeons in our center who had previous experience.

Baseline characteristics, pathological outcomes and postoperative complications were recorded. Postoperative complications were evaluated in strict accordance with the ISGPS standards [16, 17, 18].

### 1.2 Statistical Analysis

We used the computer software SPSS 26.0 for Windows (IBM Corp.) to perform all of the statistical analyses. We used medians and interquartile ranges (IQRs) or means and standard deviations (SDs) to describe continuous data, as well as numbers and percentages (%) to describe categorical data. The Mann–Whitney *U* test, Pearson's *χ* ^2^ test, Fisher's exact test, and Student's *t* test were used for data comparison. Statistical significance was defined as a *p* value < 0.05. Univariate and multivariable logistic regression analyses were performed. Two‐sided *p* values of less than 0.05 were considered to indicate statistical significance, and odds ratios (ORs) and their 95% confidence intervals (CIs) were reported. The mTO was determined for each patient, and the mTO was not achieved until all of the requirements were met. Modified TO rates were compared between the groups by using the chi‐square test.

### 1.3 Definition of Modified Textbook Outcome

The definition of the Dutch TO was based on PD and DP systems, which comprised six points. Given the differences in surgical approaches, the evaluation system is not fully applicable to pancreatic function‐preserved surgeries, such as MP. At the beginning of the study, we analyzed the data by using the Dutch TO criteria; however, the results were not as optimal as initially expected (as shown in the DISCUSSION section). Therefore, we regrouped patients and redefined the modified textbook outcomes.

The definition of mTO for MP surgery was defined as the absence of modified postoperative pancreatic fistula, postpancreatectomy hemorrhage (ISGPS grade B/C), severe complications (Clavien–Dindo ≥ III), readmission, and in‐hospital mortality. Within this, mPOPF was regarded as ISGPS B/C pancreatic fistula in combination with the following features: continuous drain flushing for more than 3 weeks, requirement for the highest‐level of antibiotic therapy (according to the WHO AWaRe system [19]) and the presence of other serious complications.

## 2 Results

### 2.1 Baseline Characteristics and Perioperative Outcomes

A total of 209 patients who underwent robot‐assisted middle pancreatectomy at our pancreatic disease center between 2010 and 2023 were included in this cohort. All of the baseline characteristics are presented in Table 1. The mean age of the patients was 45.88 ± 14.41 years, and 19.62% of the participants were elderly (≥ 60 years). Seventy percent of patients (145/209, 69.38%) were female, which suggests that female patients are more likely to develop junctional tumors such as solid pseudopapillary tumors (SPTs). The mean BMI was 22.91 ± 3.64 kg/m^2^. Only a few patients demonstrated an ASA score of III (6/209, 2.87%), and no patients had an ASA score of IV.

**TABLE 1:** Baseline characteristics and perioperative outcomes regarding mTO.

Characteristic	Total cohort (*n* = 209)	Modified textbook outcome		*p*
		Yes (*n* = 154)	No (*n* = 55)	
Age (years)	45.88 ± 14.41	45.77 ± 14.75	46.2 ± 13.54	0.849
≥ 60 years (%)	19.62	22.73	10.91	0.097
Sex				0.957
Male	64 (30.62%)	47 (30.52%)	17 (30.91%)	
Female	145 (69.38%)	107 (69.48%)	38 (69.09%)	
BMI^a^ (kg/m^2^)	22.91 ± 3.64	22.97 ± 3.43	22.74 ± 4.19	0.69
LOS^b^ (day)	18 (13)	17 (9)	34 (26)	*< 0.001*
ASA score				0.032
Level I	138 (66.03%)	94 (61.04%)	44 (80.00%)	
Level II	65 (31.10%)	55 (35.71%)	10 (18.18%)	
Level III	6 (2.87%)	5 (3.25%)	1 (1.82%)	
Operation time (min)	150 (60)	150 (60)	130 (60)	0.381
EBL (mL)	50 (50)	50 (50)	50 (70)	0.596
Pathology				0.441
Benign	188 (89.95%)	140 (90.91%)	48 (87.27%)	
Low‐grade malignant	21 (10.05%)	14 (9.09%)	7 (12.73%)	
Anastomose				*0.037*
Pan‐gas	190 (90.91%)	136 (88.31%)	54 (98.18%)	
Pan‐Jej	16 (7.66%)	15 (9.74%)	1 (1.82%)	
Unattended	3 (1.43%)	3 (1.95%)	0 (0%)	

*Note:* Italicized text indicates a statistically significant difference in *p*‐values.

Abbreviations: EBL, estimated blood loss; LOS, length of stay.

^a^ Five cases of missing data have been excluded from the statistics.

^b^ The cases of death were excluded in analyses of the postoperative LOS.

In terms of surgical outcomes, both the operation time (median 150 min) and estimated blood loss (median 50 mL) were satisfactory, and there were no differences observed between the two groups. We also categorized the patients' final pathological results and the type of anastomosis created during the operation. In the majority of patients who underwent RMP surgery, the final pathological results were consistent with the conclusions drawn from the preoperative MDT discussion. The 21 patients who were diagnosed with non‐cancerous lesions included those with intraductal papillary mucinous neoplasms (IPMNs) with high grade of dysplasia, G2‐stage pancreatic neuroendocrine tumors (pNETs), or SPTs with necrosis or mitosis figure. However, no patients with clear evidence of malignant pathology underwent this procedure. At our center, the most common type of anastomosis included pancreato‐gastric anastomosis (190/209, 90.91%), followed by pancreato‐enteric anastomosis, with very few patients (*n* = 3, 1.43%) having an unattended residual pancreatic tail. Although more patients with pancreato‐gastric anastomosis failed to achieve mTO, this result may be related to the small number of cases observed in the other groups. We observed a significant difference in the length of stay (LOS) between the two groups. Patients who achieved modified textbook outcomes exhibited a shorter postoperative hospital stay (median (IQR), 17 (9) vs. 34 (26), *p* < 0.001).

### 2.2 Modified Textbook Outcome Rates

**FIGURE 1:**
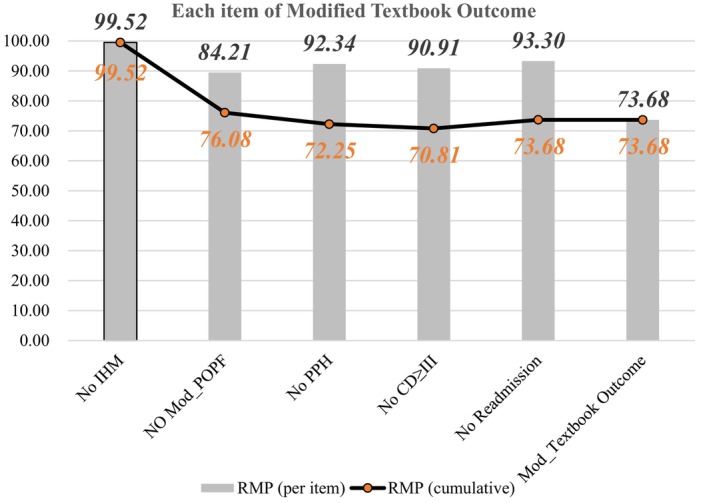
Modified textbook outcome percentages (per item and cumulative) for RMP.

In this cohort, 154 patients, that is, 73.68%, achieved modified textbook outcomes. The achievement of each of the specific points is shown in Table 2. The rates of the 5 items and the cumulative percentages are displayed in Figure 1.

**TABLE 2:** Modified textbook outcome items of RMP.

Items (*n* or %)	Total cohort (*n* = 209)	Modified textbook outcome	
		Yes (*n* = 154)	No (*n* = 55)
mPOPF^a^	33	84.21	15.79
PPH^a^	16	92.34	7.66
Complication of grade ≥ III^b^	19	90.91	9.09
Readmission	14	93.30	6.70
IHM	1	99.52	0.48

Abbreviations: IHM, in‐hospital mortality; mPOPF, modified post‐operative pancreatic fistula; POBF, post‐operation bile fistula; PPH, post‐pancreatectomy hemorrhage.

^a^ POPF, POBF, and PPH are defined by the criterion of the International Study Group of Pancreatic Fistula (ISGPF).

^b^ The classification of postoperative complications is due to Clavien–Dindo criterion.

A total of 15.79% and 33 patients were considered to have modified pancreatic fistulas, which still presented the most common post‐operative complication. The incidences of postoperative hemorrhage, serious complications (Clavien–Dindo grade ≥ III) and readmission within 30 days were similar, accounting for 7.66%, 9.09% and 6.70%, respectively.

Postoperative non‐POPF‐related hemorrhage, including abdominal and gastrointestinal bleeding, occurred in 8 patients. In addition to POPF and PPH, 3 patients experienced serious complications. A total of 14 patients were readmitted to the hospital within 30 days of discharge. Four of these patients were discharged with tubes and were readmitted due to high fever or drastic changes in the nature of the drainage fluid. The reasons for readmission for the other 10 patients included high fever, recurrent nausea and vomiting, and sudden onset of severe abdominal pain.

One patient died in the hospital after surgery. This patient was a 73‐year‐old male who experienced sudden intraductal hemorrhage on day 9. The patient was immediately transported to the operating room for digital subtraction angiography (DSA); however, no obvious vascular leakage points were observed. The next day, he again experienced bleeding and demonstrated a marked drop in blood pressure and sudden cardiac arrest; despite all medical treatment, he did not survive. The final cause of death was determined to be postoperative abdominal hemorrhage with pancreatic fistula.

### 2.3 Univariate and Multivariate Analysis

According to the univariate analysis, ASA scores II and III predicted a worse mTO rate (OR 2.14 [1.50–3.08] and 2.57 [1.24–5.79], respectively), whereas non‐cancerous lesion pathology was associated with a better mTO rate (OR 2.92 [2.12–4.09]). Multivariate analysis revealed that neither the ASA score nor the pathological outcome was significantly correlated with the mTO rate. The results of the univariate and multivariate analyses for PD and DP are presented in Table 3.

**TABLE 3:** Univariate and multivariate analysis of parameters associated with modified textbook outcome after RMP.

Clinical parameters	Univariate		Multivariate	
	OR (95% CI)	*p*	OR (95% CI)	*p*
Age (years)				
≤ 60	Ref			
> 60	2.06 (0.86–5.74)	0.1301		
Sex				
Female	Ref			
Male	0.33 (0.07–1.46)	0.1312		
ASA score				
ASA I	Ref		Ref	
ASA II	2.14 (1.50–3.08)	*< 0.0001*	1.08 (0.42–2.93)	0.9
ASA III	2.57 (1.24–5.79)	*0.0151*	1.34 (0.1–40.80)	0.8
BMI (kg/m^2^)				
< 25	Ref			
≥ 25	0.85 (0.41–1.81)	0.6583		
Operation time (min)				
< 150	Ref			
≥ 150	1.52 (0.82–2.83)	0.1864		
EBL (mL)				
< 150	Ref			
≥ 150	0.88 (0.37–2.24)	0.7710		
Pathology				
Benign	Ref		Ref	
Low‐malignant	2.92 (2.12–4.09)	*< 0.0001*	0.77 (0.23–2.86)	0.7
Anastomose				
Pan‐gas	Ref			
Pan‐Jej	5.96 (1.16–109.01)	0.0878		
Unattended	2,286,190 (0.00–NA)	0.9861		
Dilated pancreatic duct				
Yes	Ref			
No	0.94 (0.42–2.28)	0.8918		
History of diabetes				
Yes	Ref			
No	2.57 (0.44–48.64)	0.3822		
Level of glucose				
High	Ref			
Normal	1.69 (0.60–6.07)	0.3623		
Level of amylase				
High	Ref			
Normal	0.35 (0.08–1.54)	0.1496		
Level of HbA1c				
High	Ref			
Normal	0.95 (0.14–18.98)	0.9621		
Level of albumin				
Abnormal	Ref			
Normal	0.35 (0.04–2.96)	0.2981		

*Note:* Italicized text indicates a statistically significant difference in *p*‐values.

### 2.4 Trend of mTO Per Year and by Learning Curve

**FIGURE 2:**
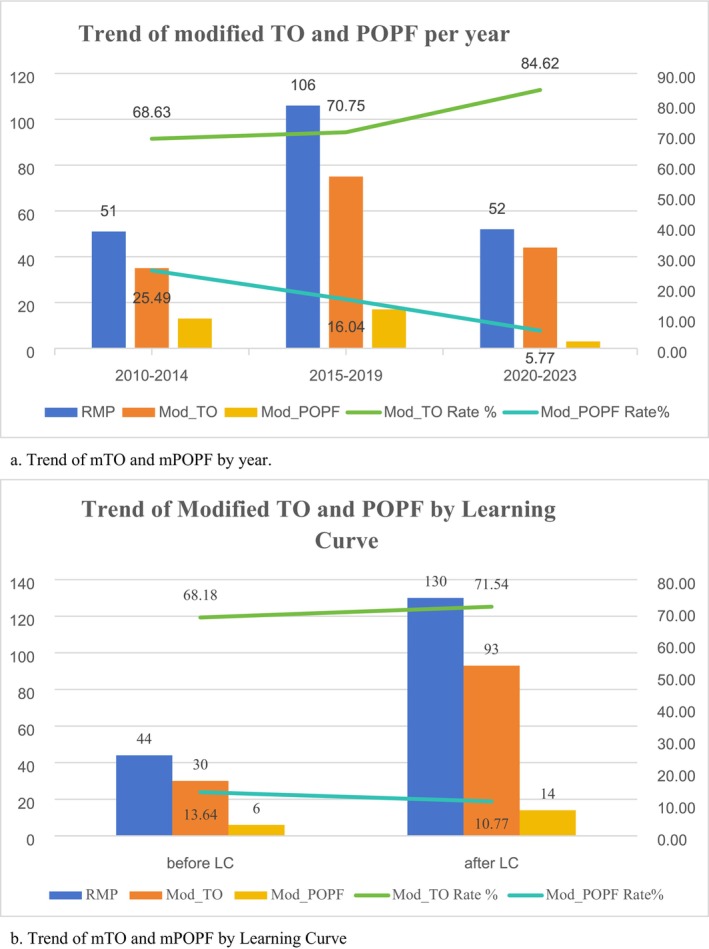
(a) Trend of mTO and mPOPF by year. (b) Trend of mTO and mPOPF by learning curve.

We calculated the incidence of pancreatic fistula and the rate of textbook outcomes by year throughout the study period. As shown in Figure 2a, the rates of mTO were 68.63%, 70.75% and 84.62% in 2010–2014, 2015–2019 and 2020–2023, respectively. The rates of mPOPF for the same time periods were 25.49%, 16.04% and 5.77%, respectively. A correlation was observed between a higher mTO rate and a lower mPOPF rate. Moreover, due to our previous study regarding learning curves, we selected all of the surgeries performed by the same surgeon for analysis. As shown in Figure 2b, after the learning curve was completed, the mPOPF rate decreased and the mTO rate increased (from 13.64% to 10.77% and from 68.18% to 71.54%, respectively).

## 3 Discussion

The concept of “textbook outcomes” was first introduced in 2013 in a study of colorectal cancer by Kolfschoten et al. [20]. A total of 5582 patients who underwent colon cancer resection at 82 hospitals in the Dutch Surgical Colorectal Audit were included in the study. The authors assessed six independent indicators of “expected results” with the all‐or‐none principle. A patient is defined as having a “textbook outcome” when he or she meets all six of these criteria. Kolfschoten et al. reported that, compared with any single indicator, the TO provides a better assessment of surgical quality, provides a simple comprehensive summary of hospital performance, and can be replicated in clinical studies to improve the overall quality of colorectal cancer surgery in the Netherlands [20]. The concept of the TO has since rapidly gained popularity in surgical research and has been advocated as being a comprehensive quality indicator for assessing complex surgical procedures, such as abdominal aortic aneurysms [21], gastroesophageal cancers [22], retroperitoneal sarcomas [23], and organ transplantation [24]. Since 2018, the TO has also been used to assess the quality of hepatobiliary and pancreatic surgery [25, 26]. In 2020, an article published in the Annals of Surgery reported the TO of pancreatic surgery, which included the absence of postoperative pancreatic fistula, bile leakage, postpancreatectomy hemorrhage (all ISGPS grade B/C), severe complications (Clavien–Dindo ≥ III), readmission, and in‐hospital mortality [4]. To obtain an international consensus on this definition, van Roessel et al. [4] conducted a survey of 24 leading pancreatic experts from 21 institutions in 10 countries on 4 continents. The survey consisted of 17 items, each of which was scored individually using a Likert scale, and items with > 80% agreement were included in the definition of a textbook outcome. Ultimately, 6 parameters were selected as previously described. Although not included as a parameter, prolonged hospitalization was analyzed as a candidate parameter in a separate subgroup of the study. In this study, 60.3% of the patients achieved the TO, with 58.3% achieving the TO after PD, and 67.4% achieving the TO after DP [4]. Although the incidence of individual complications is not high, the overall incidence of the TO remains low. These findings have been corroborated by several other studies. For example, in a study conducted by Merath et al., 47.8% of 4394 patients who underwent pancreatectomy of a small area (DP or partial pancreatectomy) achieved the TO, whereas only 24.7% of 993 patients who underwent pancreatectomy of a large area (PD or TP) achieved the TO [26]. In the study by Heidsma et al., the achievement of the TO was also associated with a better prognosis (the 3‐year DFS rate was 91.7% for patients who achieved a TO versus 85.2% for patients who did not achieve a TO, *p* = 0.003) [27]. Thus, the achievement of the TO is not only representative of the best surgical outcome but is also representative of an oncologic cure.

As a parenchyma‐sparing surgery, middle pancreatectomy (MP) can preserve normal pancreatic function to reduce the risk of exocrine and endocrine insufficiency after surgery. MP surgery, especially minimally invasive MP surgery, is undoubtedly a better option than PD or DP surgery. In the Department of General Surgery, Pancreatic Disease Center, Ruijin Hospital Shanghai Jiao Tong University School of Medicine, Shanghai, China, we conducted RMP surgery for the first time in 2010. In previous studies, we reported that compared with OMP, RMP is a shorter operation and causes less blood loss, with POPF remaining as the major complication [13]. For the RMP procedure, there were two flexion points in the learning curve were observed at procedures 12 and 44 [14]. The learning curve allows for more consistent operation times and less intraoperative bleeding. Compared with RPD, RMP demonstrates a shorter learning curve, as it is easier to optimize the surgical approach. For non‐invasive pancreatic lesions located in the neck and body of the pancreas, RMP is undoubtedly a more favorable option compared to PD or DP. Although the safety and feasibility of middle pancreatectomy have been comprehensively studied and recognized, its selection and application are still limited by the risk of a high POPF rate. Therefore, an excellent evaluation metric for determining whether middle pancreatectomy is truly worthwhile for surgeons to perform and for patients to achieve better postoperative outcomes is needed. Thus, the concept of textbook outcomes represents a promising factor. However, unlike radical procedures such as PD and DP, the criteria for TO should differ.

In the beginning of this study, we applied the TO with 6 requirements for middle pancreatectomy. Disappointingly, the TO rate was determined to only be 50.2%, which was much lower than that of both the PD and DP operations (Table S1). We believe that the use of the TO as an indicator for evaluating MP has several limitations. First, the metrics for evaluating the TO are not entirely appropriate for MPs. Bile leakage is among the 6 items of the TO, and this feature was not observed in any of the patients in our cohort. When considering the current guidelines and clinical experience, patients who are considered for MP often do not demonstrate a tumor that is adjacent to the common bile duct. This scenario makes the evaluation of “bile leakage” in MP seemingly unnecessary. Second, pancreatic fistula has a disproportionate effect on MP. Due to the nature of MP, POPF and POPF‐related complications account for the vast majority of post‐MP problems. In our cohort, only 20% (21/104) of the patients experienced non‐POPF‐related complications, whereas the overall POPF rate was close to 40% (83/209) (Table S2, Figures S1 and S2). We also reported that although MP demonstrates a very high incidence of pancreatic fistula, the proportion of grade C fistulas is lower, and the perioperative mortality rate is much lower than that of PD and DP. Moreover, we observed that there was still no method available to significantly reduce the incidence of pancreatic fistula after MP, regardless of whether the surgeons demonstrated proficiency. The learning curve is an integral part of the assessment of surgeon proficiency and reduces the operation time and intraoperative bleeding once it is mastered; moreover, it does not increase the TO rate (Figure S3). Therefore, it is possible to assess the development of POPF after MP in many additional ways other than via simple “yes” or “no” responses.

On this basis, we regrouped patients with pancreatic fistula. Specifically based on the ISGPF standard, we reclassified patients with pancreatic fistula by using the endpoint of “failure to achieve an ideal surgical outcome.” The definition of modified POPF involves the presence of an ISGPS B/C pancreatic fistula in combination with one of the following features: continuous drain flushing for more than 3 weeks, a requirement for the highest‐level antibiotic therapy, and the presence of other serious complications. In this case, the grading criteria for antibiotic therapy in this case refer to the WHO guidelines [19]. In addition, we removed “bile leakage” from the evaluation indicators. According to these 5 criteria, the overall modified textbook outcome rate increased to 73.68%. Patients achieving mTO demonstrated significantly shorter hospitalization days, and we observed that an ASA score of I and non‐cancerous lesion pathology resulted in a better mTO rate. This observation represents an encouraging result for our center. In our previous study, we successfully established a learning curve for RMP surgery. Unfortunately, even after the learning curve is passed, surgeons are still unable to avoid the high incidence of pancreatic fistula. Admittedly, years of clinical experience and subjective feelings indicated to our team that RMP surgery is worthwhile; however, no supporting evidence was available. Currently, with the establishment of the multidimensional parameter mTO, we are able to confirm our initial suspicion. After we retrospectively analyzed our data over the previous years, we also observed an increase in the mTO rate by year. An increase in the mTO rate is also noted after the learning curve is passed. These findings can provide valuable evidence for surgeons in terms of their choice of surgical approach.

The revised definition of mPOPF proposed in this study may facilitate a more accurate assessment of the clinical situation “pancreatic fistula occurs, but doesn't affect the patient's achievement of textbook outcome.” Although the incidence of pancreatic fistula following MP is higher compared to other surgical procedures, this complication alone should not preclude the use of MP when clinically indicated. In light of our center's clinical experience, we have revised the definition of mPOPF to better differentiate between clinically relevant fistulas that pose risks and those that do not adversely affect favorable surgical outcomes, thereby supporting the appropriate application of MP.

However, this indicator still demonstrates several shortcomings. Given that the patients targeted for middle pancreatectomy are people with non‐malignant tumors, the mTO definition does not contain any pathological or oncological parameters. Additionally, the existing evaluation indicators fail to represent the unique strengths of MP. MP and other function‐preserving pancreatectomies were developed to reduce the postoperative effects on organ function. In this case, there are no indicators related to long‐term functional evaluation. Patients who undergo MP exhibit benign or non‐cancerous lesions, and the impacts of these tumors on long‐term survival are relatively minor. This scenario makes the assessment of long‐term quality of life and functional retention more important. In addition, the assessment of the mTO is only developed and applied in our own center, and multi‐center clinical research is still needed to further validate and assess the feasibility and accuracy of the use of this index.

In conclusion, the mTO is an excellent evaluation metric for RMP surgery. POPF remains the most common complication of RMP; however, the safety and efficacy of RMP cannot be denied. In the future, it is necessary to explore new comprehensive indices to evaluate function‐preserved pancreatectomy including RMP.

## Author Contributions

Conceptualization: Jingfeng Li, Lihan Qian, and Yusheng Shi. Methodology: Zhiwei Xu, Xinjing Wang, Wei Xu, Xiaxing Deng, and Yusheng Shi. Software: Jingfeng Li and Lihan Qian. Data curation: Jingfeng Li, Lihan Qian, and Zhiwei Xu. Investigation: Jingfeng Li, Lihan Qian, and Zhiwei Xu. Validation: Xinjing Wang, Wei Xu, and Xiaxing Deng. Formal analysis: Jingfeng Li, Lihan Qian, and Zhiwei Xu. Supervision: Chenghong Peng, Baiyong Shen, and Yusheng Shi. Funding acquisition: Yusheng Shi. Visualization: Jingfeng Li, Lihan Qian, and Zhiwei Xu. Project administration: Chenghong Peng, Baiyong Shen, and Yusheng Shi. Resources: Chenghong Peng and Baiyong Shen. Writing – original draft: Jingfeng Li, Lihan Qian, and Zhiwei Xu. Writing – review and editing: Chenghong Peng, Baiyong Shen, and Yusheng Shi.

## Funding

National Natural Science Foundation of China (82303263).

## Conflicts of Interest

The authors declare no conflicts of interest.

## Supporting information


**Figure S1:** cam471542‐sup‐0001‐FigureS1.docx.

**Figure S2:** cam471542‐sup‐0002‐FigureS2.docx.

**Figure S3:** cam471542‐sup‐0003‐FigureS3.docx.

**Table S1:** cam471542‐sup‐0004‐TableS1.docx.

**Table S2:** cam471542‐sup‐0005‐TableS2.docx.

## Data Availability

The data that support the findings of this study are available from the corresponding author upon reasonable request.
