# Supplementary material for: Development of a Modified Textbook Outcome in Evaluating Robot‐Assisted Middle Pancreatectomy: A Real‐World Study of RMP Surgery in a High‐Volume Pancreatic Disease Center
Source: Cancer Med. 2026 Jan 30;15(2):e71542. doi: 10.1002/cam4.71542 (PMC12856511; doi:10.1002/cam4.71542)
Supplement: Supplementary file 1 — Figure S1: cam471542‐sup‐0001‐FigureS1.docx. [file CAM4-15-e71542-s004.docx]

Figure S1. Textbook Outcome percentages (per item and cumulative) for RMP.
