# Supplementary figures and images for: Development of a Modified Textbook Outcome in Evaluating Robot‐Assisted Middle Pancreatectomy: A Real‐World Study of RMP Surgery in a High‐Volume Pancreatic Disease Center
Source: Cancer Med. 2026 Jan 30;15(2):e71542. doi: 10.1002/cam4.71542 (PMC12856511; doi:10.1002/cam4.71542)

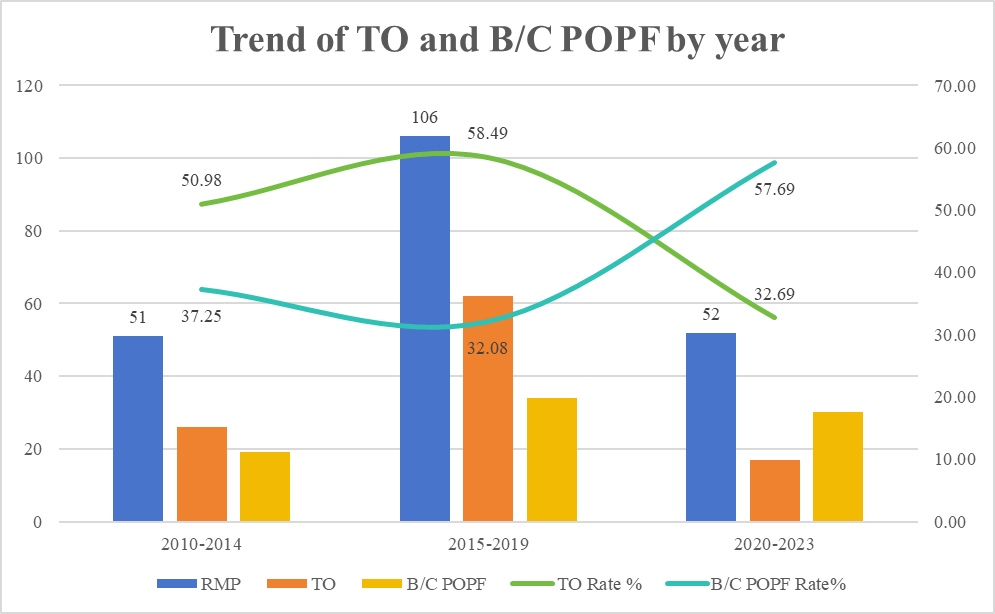


Figure S3a. Trend of TO and B/C POPF by year.


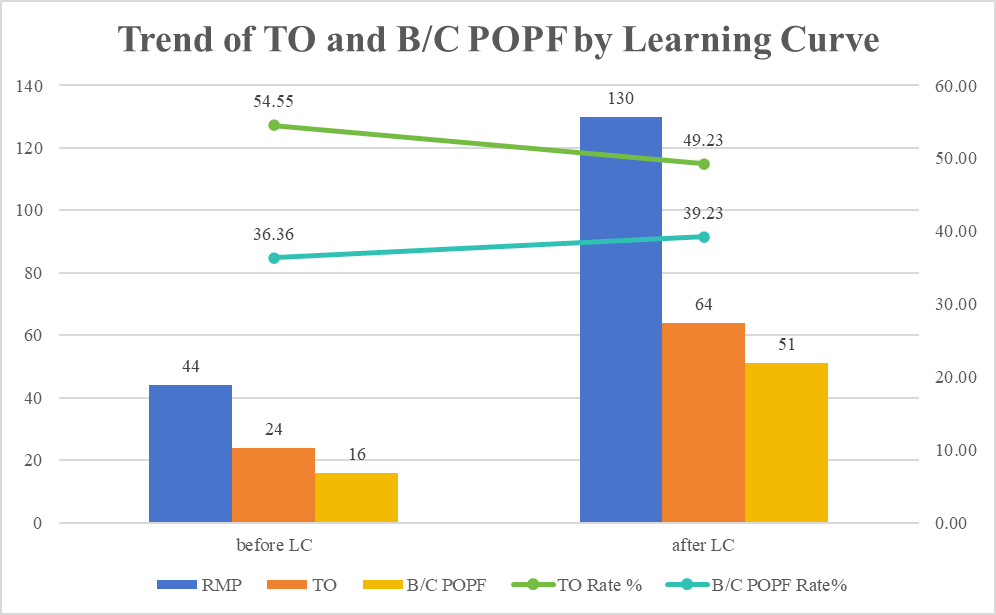


Figure S3b. Trend of TO and B/C POPF by Learning Curve

Supplement: Supplementary file 3 — Figure S3: cam471542‐sup‐0003‐FigureS3.docx. [file CAM4-15-e71542-s003.docx]
