# Supplementary material for: Development of a Modified Textbook Outcome in Evaluating Robot‐Assisted Middle Pancreatectomy: A Real‐World Study of RMP Surgery in a High‐Volume Pancreatic Disease Center
Source: Cancer Med. 2026 Jan 30;15(2):e71542. doi: 10.1002/cam4.71542 (PMC12856511; doi:10.1002/cam4.71542)
Supplement: Supplementary file 4 — Table S1: cam471542‐sup‐0004‐TableS1.docx. [file CAM4-15-e71542-s005.docx]

TABLE S1 Baseline Characteristics and Perioperative Outcomes

|  |  | | Textbook Outcome | |  |
| --- | --- | --- | --- | --- | --- |
| Characteristic | | Total Cohort  (n = 209) | Yes  (n = 105) | No  (n = 104) | *P* value |
| Age (yrs) | | 45.88±14.41 | 47.06±15.05 | 44.69±13.70 | 0.236 |
| ≥60yrs (%) | | 19.62 | 24.76 | 14.42 | 0.668 |
| Sex | |  |  |  | 0.344 |
| male | | 64(30.62%) | 29(27.62%) | 35(33.65%) |  |
| female | | 145(69.38%) | 76(72.38%) | 69(66.35%) |  |
| BMI^a^ (kg/m^2^) | | 22.91±3.64 | 22.62±3.42 | 23.18±3.83 | 0.276 |
| LOS^b^ (day) | | 18(13) | 16(11) | 21(16) | *0.001* |
| ASA score | |  |  |  | 0.151 |
| Level I | | 138(66.03%) | 74(70.48%) | 64(61.54%) |  |
| Level II | | 65(31.10%) | 30(28.57%) | 35(33.65%) |  |
| Level III | | 6(2.87%) | 1(0.95%) | 5(4.81%) |  |
| Operation Time (min) | | 150(60) | 150(60) | 150(60) | 0.403 |
| EBL (mL) | | 50(50) | 50(50) | 50(50) | 0.456 |
| Pathology | |  |  |  | 0.087 |
| Benign | | 188(89.95%) | 91(86.67%) | 97(93.27%) |  |
| Low-grade Malignant | | 21(10.05%) | 14(13.33%) | 7(6.73%) |  |
| Anastomose | |  |  |  | 0.527 |
| Pan-Gas | | 190(90.91%) | 97(92.38%) | 93(89.42%) |  |
| Pan-Jej | | 16(7.66%) | 6(5.71%) | 10(9.62%) |  |
| Unattended | | 3(1.43%) | 2(1.91%) | 1(0.96%) |  |

Abbreviations: LOS length of stay, EBL estimated blood loss

^a^Five cases of missing data have been excluded from the statistics.

^b^The cases of death were excluded in analyses of the postoperative LOS.
