# Supplementary material for: Development of a Modified Textbook Outcome in Evaluating Robot‐Assisted Middle Pancreatectomy: A Real‐World Study of RMP Surgery in a High‐Volume Pancreatic Disease Center
Source: Cancer Med. 2026 Jan 30;15(2):e71542. doi: 10.1002/cam4.71542 (PMC12856511; doi:10.1002/cam4.71542)
Supplement: Supplementary file 5 — Table S2: cam471542‐sup‐0005‐TableS2.docx. [file CAM4-15-e71542-s002.docx]

TABLE S2 Textbook outcome items of RMP

|  |  | | | Textbook Outcome | |
| --- | --- | --- | --- | --- | --- |
| Items (n or %) | | Total Cohort  (n = 209) | | Yes  (n = 105) | No  (n = 104) |
| CR_POPF^a^ | | | 83 | 60.29 | 39.71 |
| Grade B | | | 80 |  |  |
| Grade C | | | 3 |  |  |
| POBF^a^ | | | 0 | 100.00 | 0.00 |
| PPH^a^ | | | 16 | 92.34 | 7.66 |
| POPF-related | | | 8 |  |  |
| Non-POPF-related | | | 8 |  |  |
| Complication of grade≥III^b^ | | | 19 | 90.91 | 9.09 |
| POPF-related PPH | | | 8 |  |  |
| Non-POPF-related PPH | | | 8 |  |  |
| Else | | | 3 |  |  |
| Readmission | | | 14 | 93.30 | 6.70 |
| POPF-related | | | 4 |  |  |
| Non-POPF-related | | | 10 |  |  |
| IHM | | | 1 | 99.52 | 0.48 |

Abbreviations: CR_POPF clinical related post-operative pancreatic fistula, POBF post-operation bile fistula, PPH post-pancreatectomy hemorrhage, IHM in-hospital mortality.

^a^POPF, POBF and PPH is definded by criterion of the International Study Group of Pancreatic Fistula (ISGPF).

^b^The classification of postoperative complications is due to Clavien-Dindo criterion.
